# Elevating the Role of the Outdoor Environment for Adolescent Wellbeing in Everyday Life

**DOI:** 10.3389/fpsyg.2022.774592

**Published:** 2022-03-03

**Authors:** Mark Wales, Fredrika Mårtensson, Eva Hoff, Märit Jansson

**Affiliations:** ^1^ Department of People and Society, Swedish University of Agricultural Sciences (SLU), Alnarp, Sweden; ^2^ Department of Psychology, Lund University, Lund, Sweden; ^3^Department of Landscape Architecture, Planning and Management, Swedish University of Agricultural Sciences (SLU), Alnarp, Sweden

**Keywords:** public open space (POS), urban planning and design, adolescent development, youth-friendly environments, environmental psychology, salutogenic affordances, independent mobility, ecological systems approach

## Abstract

In light of concerns about adolescent mental health, there is a need to identify and examine potential pathways to wellbeing in their daily lives. Outdoor environments can offer multiple pathways to wellbeing through opportunities for restoration, physical activity and socialising. However, urbanisation and new lifestyles revolving around the home and the internet are changing young people’s access, use and relationship to the outdoor environment. The authors point out how the research related to adolescents’ outdoor environments is generally not treated with the same level of importance or as comprehensively as that for younger children. The aim of this paper is to pave the way for research and planning initiatives on everyday outdoor environments promoting the wellbeing of adolescents and the authors suggest ways in which perspectives from developmental psychology might inform the study of adolescents’ outdoor environments. The paper concludes by calling for an elevated focus on the role of outdoor environments in adolescents’ everyday lives as a source of wellbeing and more research that makes clear the specific attributes, activities and experiences related to places outdoors which make adolescents feel good.

## Introduction

Mental health problems among adolescents appear to be increasing on a global scale (Collishaw, 2015; Patton et al., 2016; Patalay and Gage, 2019). This worrying trend is attributed to circumstances in family, school and everyday life linked to globalisation, urbanisation, digitalisation and environmental degradation (Tomasik et al., 2012; Collishaw, 2015; Patton et al., 2016). More recently, new routines established during the COVID-19 pandemic, such as online teaching and other social distancing measures, have posed further challenges (Guessoum et al., 2020; Magson et al., 2021). In light of this it becomes urgent to identify possible pathways to mental health and wellbeing in the everyday lives of adolescents, also the foundation for wellbeing during adulthood (World Health Organization [WHO], 2004; Patton et al., 2016).

Environment-based approaches to improve health and wellbeing are acknowledged to offer more encompassing and long lasting effects than many individual-based measures (Ward Thompson, 2013). Outdoor environments in particular house many vital everyday activities that offer pathways to adolescent wellbeing by serving social life, nature contact and other recreational activities (Knöll and Roe, 2017; Owens, 2020; Mygind et al., 2021). However, many young people spend much time indoors in sedentary activity with negative consequences for their wellbeing (Hoare et al., 2016; Oswald et al., 2020). While the provision of salutogenic (i.e., promoting health and wellbeing) and child-friendly outdoor (play) environments for younger children has caught considerable attention in research, policy and planning (Chawla, 2015; Wells et al., 2018; UNICEF, 2019; Clark et al., 2020), the topic has not been treated with the same level of importance, detail or care in relation to adolescents.

We urge for an effort to pinpoint the distinctive role of outdoor environments in the context of adolescents’ everyday lives, taking on the challenge to map potential pathways to adolescent wellbeing for which their relationship to place is vital. For the purposes of this article the term wellbeing encompasses various aspects of emotional, psychological and social wellbeing (Keyes, 2006) and our understanding of development begins with Bronfenbrenner’s ecological systems theory as a foundation to build on and understand adolescent wellbeing in the context of place. As to what constitutes the “outdoor environment,” this is an empirical question, as they are the outdoor spaces where adolescents spend time. This includes any gardens at home, parks, playgrounds and other outdoor facilities in the neighbourhood, their school grounds, but also the streets, squares and any surrounding landscape accessible to them, such as forests, lakes or beaches.

## Place and Adolescent Wellbeing

Adolescence is a distinct period of life between childhood and adulthood that begins with puberty and spans roughly 10–19 years old (World Health Organization [WHO], 2015), although research suggests this period may in fact last until 24–25 years old (Sawyer et al., 2018). This maturational period is characterised by rapid and profound physical, cognitive, social and psychological changes that are pivotal for the life course (Dahl et al., 2018).

Individuals’ repeated interactions with their immediate physical and social surroundings over time fuel their development and are profoundly formative (Bronnfenbrenner and Morris, 2006). For example, the gradual attainment of independence and autonomy during adolescence builds on prior childhood experiences (Dahl et al., 2018) that are the function of the individual’s characteristics, their family, their living environment, and the society in which they live (Bronnfenbrenner and Morris, 2006). Parents often orchestrate children’s access to salutogenic environments. With repeated visits to places such as playgrounds, children work on their independent mobility; that is their ability to move around freely outside without adult supervision (Wales et al., 2020). Through their growing independent mobility children are able to take advantage of the affordances, or the perceived function (Heft, 1988), of the outdoor environment. These two factors form the foundation of a child-friendly environment (Kyttä, 2004). By adolescence individuals have the knowledge, confidence and networks to extend their range of movement, pursue their own interests and create and maintain place attachments and social relationships crucial for their development and wellbeing (Horton et al., 2013; Arvidsen and Beames, 2018; Cox, 2020).

Adolescents’ ability to realise their new found autonomy and find socially meaningful places are vital parts of a youth-friendly environment (Lopes et al., 2018). It should be noted, however, that this should not be taken for granted nor does it occur automatically. It is a result of a complex web of arrangements between adolescent, parent and their everyday environment. Its significance for young people’s ability to promote their own wellbeing should not be understated and it is essential that spatial practitioners, such as planners and landscape architects, are well-informed (Arvidsen and Beames, 2018). Independent mobility differs between genders (Christensen and Mikkelsen, 2013; Schoeppe et al., 2016), abilities (Bedell et al., 2013) and living environments (Veitch et al., 2017). Studying adolescents’ independent mobility and mobility patterns can help us detect the role of the physical and social environment as part of a larger network of people, places and objects supporting adolescent wellbeing.

It has been argued that congruity, or a positive relationship, between individual and living environment, is the very foundation of wellbeing (Horelli, 2006; Moser, 2009). When there is a “good fit” between the two, this is revealed through an individual’s positive perceptions of the particular environment (Uzzell and Moser, 2006). Accordingly, it is likely that youth try to spend time in and bond to places which possess characteristics that mirror their developmental needs (Clark and Uzzell, 2006; Korpela, 2012). Adolescents’ own evaluations and perceptions of their lives (Lippman et al., 2011; Navarro et al., 2015) and living environments (Travlou et al., 2008; van der Burgt, 2013; Lopes et al., 2018) are therefore vital for understanding how a particular place facilitates their ability to meet their needs.

Adolescents’ needs and aspirations stem from developmental changes connected to the onset of puberty, as well as structural and functional changes to the brain, that emerge through their growing interest in thrill-seeking, peers and their wider social context (Dahl et al., 2018). Owens (2020) draws on developmental and environmental psychology in describing how place helps adolescents solve various developmental tasks pertinent to adolescence and describes how the public realm can help youth to nurture social relationships, manage free time and stimulate self-reflection. Korpela (1992), p. 251 describes how “contexts deliberately chosen or shaped by the individual deserve particular attention because they may form a major strategy in the service of development.” Indeed, it is during adolescence we acquire the ability to “adaptively pursue new goals and priorities” (Dahl et al., 2018, p. 442), making adolescents more than just “passive targets of environmental influences” (Salmela-Aro, 2010, p. 14).

## Outdoor Pathways to Wellbeing

The literature describes how outdoor environments can provide multiple pathways to wellbeing (Hartig et al., 2014; Kyttä and Broberg, 2014; Fleckney and Bentley, 2021), helping to reduce harm, but also serving to build and restore various capacities (Markevych et al., 2017). We identify three pronounced pathways in relation to adolescents; the restorative nature, physical activity and social life.

Natural environments have documented benefits for adolescent emotional, psychological, and social wellbeing (Chawla, 2015; Tillmann et al., 2018; Vanaken and Danckaerts, 2018; Wells et al., 2018) and there are studies documenting associations with adolescents’ access, exposure and engagement with nature (Mygind et al., 2019; Zhang et al., 2020). Other studies improve our understanding on how and why they actively seek out natural spaces and describe how they can provide a feeling of calm and of getting away as well as a safe environment in which to be and find oneself (Birch et al., 2020; Hakoköngäs and Puhakka, 2021). Different pathways to wellbeing can occur at different levels of interaction, ranging from indirect engagement when looking at some trees through a window, to more incidental engagement when passing a park on the way to school, to more purposive use when playing sports (Pretty, 2004). For example, a study from Finland revealed girls aged 13–16 visited nature to experience pleasant emotions, be active and feel better (Wiens et al., 2021). Other nature-based activities, such as wilderness therapy and outdoor education are also used to treat mental health problems, boost self-esteem and enhance learning (Barton et al., 2016; Mutz and Muller, 2016; Manner et al., 2020). In contrast to this, a recent study revealed how everyday, more urban nature was often more valued by youth than more rural, activity-based nature experiences (Birch et al., 2020). More detailed research is needed to reveal how different kinds of nature and activities promote different dimensions of wellbeing for different people.

Despite adolescence being a period of declining physical activity (Bélanger et al., 2019), exercise is one of the main reasons for youth to venture outside (Lopes et al., 2018; Hakoköngäs and Puhakka, 2021; Wiens et al., 2021) and physical activity generally increases outdoors (Dunton et al., 2007; Pagels et al., 2014; Bélanger et al., 2019). This makes it an important mediator between time spent outdoors and wellbeing. For youth the presence of paths, proximity to parks, playgrounds and sport facilities, traffic safety and an overall varied landscape, are some of the factors triggering physical activity (Gardsjord et al., 2014; Johansson et al., 2020). School ground greening has also been linked to wellbeing through improved opportunities for physical activity, but also mental restoration with implications for attention in class and school achievement (Chawla et al., 2014; Mårtensson et al., 2014; Kelz et al., 2015; Jansson et al., 2018).

When entering adolescence the social aspects of outdoor life gain extra importance and places are often valued by adolescents in terms of the presence and/or absence of others (Clark and Uzzell, 2006; Travlou et al., 2008; Owens, 2020). For example, outdoor settings are often chosen by adolescents to hang out with friends away from the parental gaze. The dominance of the social in outdoor life is exemplified by Portuguese adolescents who marked more social affordances than leisure, emotional or functional (play) affordances in a neighbourhood mapping exercise (Lopes et al., 2018). Through their social interactions in the neighbourhood adolescents develop a sense of belonging and become part of a community which is formative for their identity and contributes to their psychological wellbeing (Morrow, 2000; Matthews, 2003; Barron, 2021).

In summary, research has documented how the social nature of adolescence means the value of outdoor environments is often understood in relation to others, making them heavily social environments, but also settings for restoration and recreation (Korpela et al., 2002; Owens, 2009; Brunelle et al., 2018). One study describes how children under 11 years old use outdoor space as a setting for play and games, 13 year olds as a place for hanging out and be “where things happen,” and older youth as a place to get away from the hassles of daily life (Matthews, 2003). Adolescents have also been shown to show lower emotional connection to nature than younger children, with a low point at 15–16 years old (Hughes et al., 2019). On the other hand, adolescents regularly list their favourite places as being in natural environments when asked (Owens and McKinnon, 2009; La Rochelle and Owens, 2014; Birch et al., 2020). There is a research gap with regards the similarities and differences in dimensions of outdoor life that are essential across the lifespan from childhood to adulthood.

## Discussion

In this paper we have highlighted the role of outdoor environments in adolescents’ everyday lives and pointed out how by scrutinising the interplay between the two as development embedded in social and physical contexts we can improve our capacity to create youth-friendly environments which promote their wellbeing. However, adolescents’ ability to take advantage of their growing role as active agents of their own wellbeing is circumscribed by the societal context in which they live (Bronnfenbrenner and Morris, 2006; Broberg et al., 2013).

The way in which society perceives adolescents has consequences for their wellbeing. Conceptions of adolescents in public spaces as being at risk and/or problematic are common (Travlou, 2003) and have repercussions for adolescents’ ability to exercise their autonomy and find places that fit their needs. Adolescents can be viewed suspiciously, made to feel unwelcome and even excluded from spaces through spatial practices (i.e., planning, design and management) that restrict their activities (Owens, 2002; Woolley et al., 2011). Where adolescents are allowed to enter school grounds at night, they tend to become favourite hang-outs as they provide a sense of security and belonging, as well as privacy. They might play music while they talk and swing and smoke. The lack of supervision and their behaviour is often negatively interpreted (Owens, 2020), but research suggests such behaviour is a complex issue which for the adolescent fill an important function for self-regulation (Ward Thompson et al., 2005). Other people’s perceptions can influence whether or not they feel welcome and hinder their ability to have meaningful experiences outdoors that are central to the quality of youth-friendly environments (Broberg et al., 2013; Lopes et al., 2018).

The way adolescents are perceived in spatial practices influences the outdoor environments adolescents have access to. Perceptions of them as competent and autonomous appear to have placed much of the responsibility on adolescents’ themselves to meet their place needs through their appropriation of space in other people’s places (Childress, 2004). This is further reflected in the growing focus on youth participation in spatial practices (Bishop and Corkery, 2017; Derr et al., 2018; Loebach et al., 2020). The agency of youth in spatial practices is a truly vital aspect of their wellbeing, but should not get mixed up with the overarching responsibility of adults having to make decisions in their best interest (Vanderbeck, 2008). In contrast, the perception of (younger) children as less competent and more vulnerable, has instilled a sense of duty among adults to provide playgrounds, an infrastructure recognised as an essential part of public space in many parts of the world (Jansson, 2010; Woolley and Lowe, 2013).

Outdoor spaces specifically allocated for adolescents are rare (Owens, 2017; Sundevall and Jansson, 2020) and the unique experiences of adolescents have not received the attention they deserve, resulting in a neglect of adolescents’ place needs. Valentine (2019) suggests this stems from a view of adolescents as problematic and confusion surrounding definitions of “adolescents,” “youth,” and “teenagers” which has meant the study of adolescents’ relationship with place is regularly engulfed by the field of children’s geographies. As a result, the study of youth geographies lacks its own identity as a field for practice and research. While the distinctive features of child development are regularly taken into account in playground design, little attention is paid to the unique characteristics of adolescence in spatial practices (Owens, 2020). Maybe the focus on play in children’s outdoor behaviours is easier (and more desirable) to plan for than the more complex (and problematic) behaviours of adolescents outdoors? We argue a discourse preoccupied with the salutogenic effects of nature and the dominance of the social features of adolescents’ outdoor lives has refrained us from better harnessing the potential of adolescents’ everyday outdoor environments. It may also mean other aspects of value for their wellbeing might be overlooked, such as their urge for independent mobility (Arvidsen and Beames, 2018), their need for places to be alone (Clark and Uzzell, 2006) and their desire to play (Ward Thompson, 2007; Owens, 2018). If we ask them, just like children, adolescents also describe environmental qualities and places that they like, need and aspire to visit (Jansson et al., 2018; Owens, 2018; Van Hecke et al., 2018).

## Conclusion

In this paper we have examined the role of outdoor environments for adolescent wellbeing and illustrated some of the pathways through which it can support and promote wellbeing; the most pronounced being restorative nature experiences, physical activity and social opportunities. We have shown how adolescents actively contribute to their own wellbeing through selecting environments that fit their needs and aspirations. We have also highlighted how important the increase in independent mobility actually is in the transition from childhood to adolescence in their continued development. Some of the societal influences limiting adolescents’ ability to take full advantage of the salutogenic potential of outdoor environments have also been discussed. Misleading preconceptions about adolescents and their behaviour in outdoor environments prevail. These have to be contested! Moreover, we point out how there is an imbalance in the emphasis placed on the social nature of adolescents’ lives and the role of nature in contrast to other key aspects and the specificities of the physical contexts of their everyday outdoor lives. With examples from research literature across urban and rural conditions we have illustrated how intertwined the activating, social and restorative roles of the outdoor environment can be in the daily life of adolescents. This makes it hard to identify the full range of pathways and benefits for adolescents themselves. We argue that by adopting a developmental approach to the study of adolescents’ outdoor lives, as a complement to the existing body of research, we can make the benefits more transparent for society and spatial practictioners and create more youth-friendly environments.

Considering the current state of adolescent mental health, it is therefore time for research and spatial practices to further elevate the role of outdoor environments in the service of adolescent wellbeing. In order to do this and actualise the salutogenic potential of outdoor environments we suggest researchers and spatial practitioners address the following four challenges:

(1) Identify the full range of outdoor environments and experiences which comprise adolescents’ everyday lives.
(2) Characterise the specificities of adolescents’ outdoor lives and the attributes of outdoor environments which support their wellbeing. Particular attention needs to be paid to the (often neglected) specific physical characteristics which help to create youth-friendly environments.
(3) Link findings on adolescents’ outdoor lives and place preferences to the growing body of research on adolescent development and wellbeing. Focus should be on revealing, understanding and making transparent the different pathways to wellbeing which exist.
(4) Follow adolescents’ outdoor lives over time to reveal the nuances and value of their outdoor experiences throughout adolescence and how they develop over time, from early (10–14 years old) to late adolescence (15–19 years old), as well as across seasons.

## Data Availability Statement

The original contributions presented in the study are included in the article/supplementary material, further inquiries can be directed to the corresponding author.

## Author Contributions

MW conceived the idea for the manuscript. All authors contributed to the writing and development of the manuscript’s ideas and read, and approved the final manuscript.

## Conflict of Interest

The authors declare that the research was conducted in the absence of any commercial or financial relationships that could be construed as a potential conflict of interest.

## Publisher’s Note

All claims expressed in this article are solely those of the authors and do not necessarily represent those of their affiliated organizations, or those of the publisher, the editors and the reviewers. Any product that may be evaluated in this article, or claim that may be made by its manufacturer, is not guaranteed or endorsed by the publisher.
